# Antimicrobial stewardship programs in Italy: structure, process and outcome indicators, 2017-2019

**DOI:** 10.1093/eurpub/ckac131.387

**Published:** 2022-10-25

**Authors:** C Vicentini, V Blengini, G Libero, R Raso, CM Zotti

**Affiliations:** 1 Department of Public Health, University of Turin, Turin, Italy; 2 SEREMI, ASL AL, Alessandria, Italy

## Abstract

**Background:**

The increasing spread of antimicrobial resistance (AMR) represents a global public health threat, and a significant challenge for healthcare delivery. Antimicrobial stewardship (AMS) programs have proven to be effective and cost-effective strategies for optimizing antimicrobial use. We aimed to assess key aspects of AMS programs implemented in acute-care trusts of the region of Piedmont, and to evaluate changes in antimicrobial usage against a score we attributed to structural and functional elements of AMS programs.

**Methods:**

AMS programs operating in acute-care trusts in the region of Piedmont were investigated via a survey addressing program characteristics, divided into structure and process quality indicators. All public health trusts of the region of Piedmont were invited to complete the survey. The indicators were selected based on core elements identified by international guidelines and were reviewed by a multi-disciplinary panel. Antimicrobial usage was expressed as defined daily doses, DDD per 1000 patient-days. The annual means for the years 2017-2019 were considered, as well as the percentage change between 2017 and 2019. Variables were investigated in relation to structure and process scores using Spearman correlation. Analyses were performed using SPSS v. 27.0 (SPSS Inc., Armonk, NY).

**Results:**

In total, 25 AMS programs were surveyed. Higher scores were achieved for process rather than structure indicators. Improvements in total antimicrobial usage (-4%) were found between 2017 and 2019. A moderate correlation was found between structure score and percentage change in antimicrobial usage (Spearman’s ρ -0.603, p 0.006).

**Conslusions:**

This study highlighted important areas for improvement, such as accountability, microbiological laboratory quality management and feedback. Repeated measurements of structure, process and outcome indicators will be important to guide continuing quality improvement efforts.

**Key messages:**

• Results of this study support the effectiveness of AMS programs in reducing antimicrobial use.

• Important areas for improvement were identified. Improving the organization of AMS programs in particular should be prioritized.

